# Prevalence and associated factors of low resilience among public primary healthcare workers in Kuala Lumpur: A cross-sectional study

**DOI:** 10.51866/oa.944

**Published:** 2026-05-18

**Authors:** Azidah Abdul Kadir, Kaniswari Munusamy, Aida Syaqilla Bee Mohd Yusoff, Shahirul Farhanim Abd Rahman, Anesh Govindnair, Peiyi Hong, Suhaiza Samsudin

**Affiliations:** 1 Department of Family Medicine, School of Medical Sciences, Health Campus, Universiti Sains Malaysia, Kubang Kerian, Kelantan, Malaysia.; 2 Poliklinik Warga, Tingkat 1, Bangunan Klinikal, Hospital Canselor Tuanku Muhriz, Jalan Yaacob Latif, Bandar Tun Razak, Wilayah Persekutuan Kuala Lumpur, Malaysia.; 3 Klinik Keluarga Desa Tun Hussein Onn, Jalan Jelatek, Kuala Lumpur, Malaysia.; 4 Klinik Kesihatan Cheras, Jalan Yaacob Latif, Cheras, Kuala Lumpur, Malaysia.; 5 Klinik Seri Indah, No 30, Jalan 1/42, Taman City, W.P Kuala Lumpur, Malaysia.; 6 Klinik Medipulse Bukit Jalil, No. 52 G, Jalan 17/155C, Bandar Bukit Jalil, Kuala Lumpur, Malaysia.; 7 Department of Family Medicine, Kulliyyah of Medicine, International Islamic University Malaysia, Kuantan Pahang, Malaysia.

**Keywords:** Resilience, Psychological, Primary healthcare, Health personnel

## Abstract

**Introduction:**

Primary healthcare systems face many challenges, making it imperative to assess the resilience of primary healthcare workers (HCWs). This study aimed to uncover the prevalence and associated factors of low resilience among HCWs in W.P Kuala Lumpur.

**Methods:**

A cross-sectional study was conducted among 430 HCWs in public primary healthcare facilities in W.P Kuala Lumpur. A sociodemographic questionnaire, the Patient Health Questionnaire-9, the General Anxiety Disorder-7 and the 25-item Wagnild–Young Resilience Scale were administered.

**Results:**

Four hundred thirty HCWs were approached, among whom 415 were included in the analysis (response rate=96.5%). The prevalence of low resilience was 31.6%. Female sex (odds ratio [OR]=1.87; 95% Confidence Interval [CI] =1.05–3.32), ≤10 years of work experience (OR=1.94; 95% CI=1.20–3.16) and depression (OR=2.53; 95% CI=1.46–4.41) were associated with low resilience.

**Conclusion:**

The high prevalence of low resilience among primary HCWs is a critical concern. Factors such as being a woman, having limited work experience and experiencing depression are associated with low resilience. It is imperative to identify and implement targeted interventions to promote resilience among HCWs effectively.

## Introduction

Resilience can be defined as the dynamic process through which an individual demonstrates the capacity to positively adapt to challenging life experiences by exhibiting mental, emotional and behavioural flexibilities in response to both external and internal demands.^1^ Healthcare workers (HCWs) exhibiting high levels of resilience are adept at upholding uninterrupted healthcare services and mitigating burnout while preparing for disasters and managing the risks they pose.^2^ The capacity for resilience is paramount, particularly for HCWs, as it enables them to effectively adjust, positively adapt and recuperate from adversities while consistently confronting challenges.

In a meta-analysis of27,720 studies across 16 countries, the prevalence of low resilience was reported to be 26%.^1^ Similarly, the recent meta-analysis by Janitra et al.^3^ involving 51,119 participants showed an overall prevalence of low resilience of 27.0% (95% CI=21.0–33.0). Specifically, the general population demonstrated a prevalence of 35.0% (95% CI=28.0-42.0), while health professionals exhibited a prevalence of 23.0% (95% CI=16.0–30.9).^3^

Multiple factors are associated with low resilience among HCWs. While relevant studies exist, there is no consensus on the factors influencing resilience. A study conducted in Italy found that female HCWs had lower resilience than male HCWs, likely due to greater emotional stress and physical exhaustion in their caregiving roles.^4^ Another study performed in Portugal revealed that doctors tended to be more resilient than nurses, possibly due to the nature of their work. Nurses’ closer relationships with patients relative to their problems and anxieties could weaken their resilience.^5^ Additionally, a study conducted in Türkiye found that physicians with 15 years of experience exhibited greater psychological resilience during the COVID-19 outbreak. The same Turkish study also revealed that HCWs with underlying depression had a significantly higher incidence of low resilience.^6^

The growing emphasis on resilience in the workplace, particularly concerning staff retention, underscores the need to delve into the concept of resilience among healthcare professionals in the primary care setting. In Malaysia, primary HCWs are confronted with the daunting tasks of delivering comprehensive care to a sizable population, prioritising preventive and promotive measures and addressing a wide array of health concerns at the community level. The burden on primary HCWs encompasses managing the escalating prevalence of chronic diseases such as obesity, diabetes and hypertension, in addition to tackling undiagnosed cases of these conditions. Furthermore, primary HCWs are entrusted with the crucial role of ensuring seamless coordination and continuity of care for patients with complex health conditions requiring tertiary care.

HCWs are often confronted with markedly challenging work situations, making healthcare one of the most demanding professions. However, there has been a lack of focus on addressing burnout, resilience and quality of life among Malaysian HCWs compared with foreign workers. It is essential to identify and address low resilience among primary HCWs in Malaysia to alleviate the burden on the healthcare system and account for cultural differences.

The impact of low resilience on HCWs has been well documented in previous studies conducted in foreign countries. However, there is a noticeable gap in research regarding identifying the specific factors contributing to low resilience among primary HCWs in Kuala Lumpur. This study seeks to address this gap by determining the prevalence and associated factors of low resilience among primary HCWs in Kuala Lumpur. The results of the study have the potential to inform the development of targeted guidelines aimed at bolstering resilience and ultimately enhancing the quality of primary healthcare services.

## Methods

### Study design and population

This cross-sectional study was conducted among primary HCWs in Kuala Lumpur from 1 July 2023 to 30 September 2023. Through cluster random sampling, eight clinics across four administrative zones were selected, and all HCWs in these clinics were enrolled. Public primary HCWs in Kuala Lumpur aged 20–60 years were included in the study, while private primary HCWs and those on leave were excluded. The sample size was calculated using the single-proportion formula, assuming a prevalence of 50% and an accuracy of 0.05.^7^ Given a 10% non-response rate, the estimated total sample size required was 423 participants.^18^

### Data collection

Data were collected via face-to-face methods, and questionnaires were self-administered. Eligible HCWs were approached and screened according to the inclusion and exclusion criteria to determine eligibility for participation. Those who met the inclusion criteria were provided with an explanation of the study and reassured that all information gathered will be kept confidential. Participation was voluntary, and anonymity was preserved. When participants agreed, they needed to read and sign the informed consent form. Guided self-administered questionnaires were distributed, and the researchers were available to provide guidance, when needed.

### Study instruments

A sociodemographic questionnaire, the Patient Health Questionnaire-9 (PHQ-9)^16^, the General Anxiety Disorder-7 (GAD-7)^17^ and a validated Malay version of the 25-item Wagnild–Young Resilience Scale^10^ were utilised. The sociodemographic questionnaire consisted of questions on age, sex, race, educational level, marital status, number of children, occupation, duration of work experience, experience in managing patients with COVID-19, comorbidities, history of underlying psychological condition (depression/anxiety), organisational factors and perception of gratitude from the public/patients.

The 25-item Wagnild–Young Resilience Scale, developed in 1988, is a validated tool widely used to measure resilience across diverse populations. It boasts a high reliability index, with an alpha coefficient of 0.91. Each item is scored on a 7-point scale ranging from 1 *(strongly disagree)* to 7 *(strongly agree).* The total possible score ranges from 25 to 175. Individuals scoring below 120 are considered to have low resilience.^10^

The PHQ-9 is used for rapid identification of individuals with major depressive disorder and evaluation of the severity of their depressive symptoms.^16^ This widely used screening tool consists of nine questions, with each response being assigned a score ranging from 0 to 3. The total score is then calculated by adding up the scores for all questions. Scores of 0–4 suggest the absence of symptoms or the presence of minor symptoms not indicative of depression. Scores of 5–9 indicate mild depressive symptoms. Scores of 10–14 point to moderate symptoms that may benefit from treatment. Scores of 15–19 signify moderate to severe symptoms necessitating treatment. Scores of 20 and above represent severe symptoms requiring immediate treatment.^16^

The GAD-7 is a valuable tool used in screening generalised anxiety disorder in primary care. 17 With seven questions and a scoring system ranging from 0 to 3 for each response, it provides a clear indication of anxiety levels. Scores of 0–5 suggest mild anxiety; 6–10, moderate anxiety; 11–15, moderately severe anxiety; and 15–21, severe anxiety.^17^

Work experience was defined as the total number of years working in the healthcare sector. Team building referred to the availability of regular team-building activities organised by the organisation. Comorbidity was described as self-reported medical illness. The lack of leader appreciation was evaluated using a question asking whether HCWs felt that their supervisors/leaders appreciated the work they do. Conversely, leader support was assessed using a question about whether HCWs felt that their supervisors/leaders supported them.

### Data analysis

Data entry and statistical analysis were performed using the IBM SPSS Statistics for Windows, version 26.0 (IBM Corp., Armonk, NY, USA). Descriptive statistics were employed to summarise the sociodemographic characteristics of participants. Categorical data were presented as frequencies and percentages and numerical data as means and standard deviations (when normally distributed).

Multiple logistic regression analysis was employed to identify the factors associated with low resilience. Simple logistic regression was used to select variables for the multiple logistic regression analysis. Variables with P-values of <0.250 in the simple logistic regression were included in the multiple logistic regression. Backward and forward stepwise methods were used for variable selection. All possible two-way interactions were checked, and significant variables were included in the model. The independent variables were entered into the multiple logistic regression, and variance inflation factors were calculated to assess multicollinearity. The model’s fitness was assessed using the Hosmer– Lemeshow goodness-of-fit test, a classification table and receiver operating characteristic curves. Adjusted odds ratios (ORs) were estimated with their corresponding 95% CIs. The final model was then presented with adjusted ORs, 95% CIs, Wald statistics and P-values. A P-value of <0.050 was set as the level of significance.

## Results

Of the 430 respondents, 15 (3.5%) were excluded due to missing data, yielding a response rate of 96.5% (415 participants). The average resilience score among the participants was 132.7 (18.7), while the prevalence of low resilience was 31.6%. Table 1 presents the sociodemographic, work and psychological characteristics among the low and high resilience groups (N=415).

**Table 1:** Sociodemographic, work and psychological characteristics among the low and high resilience groups (N=415).

Variable	Total	Low resilience (n=131)	High resilience (n=284)	P-value
		n (%)	n (%)	
**Age (year)**				
20–40	361	117 (89.3)	244 (85.9)	0.56
41–60	54	14(10.7)	40 (14.1)	
**Sex**				
Male	98	23 (17.6)	75 (26.4)	0.05
Female	317	108 (82.4)	209 (73.6)	
**Ethnicity**				
Malay	318	97 (74.0)	221 (77.8)	0.60
Chinese	32	12 (9.2)	20 (7.0)	
Indian	44	15 (11.5)	29 (10.2)	
Others	21	7 (5.3)	14 (4.9)	
**Marital status**				
Single/divorced/widowed	124	38 (29.0)	86 (30.3)	0.85
Married	291	93 (71.0)	198 (69.7)	
**Educational level**				
Diploma or lower	248	83 (63.4)	165 (58.1)	0.090
Bachelor’s degree or higher	167	48 (36.6)	119 (41.9)	
**Number of children**				
0	226	78 (59.5)	148 (52.1)	0.80
1–3	99	26 (19.8)	73 (25.7)	
4–6	66	23 (17.6)	43 (15.1)	
7–9	24	4 (3.1)	20 (7.0)	
**Occupation**				
Medical officer	94	24 (18.4)	70 (24.6)	0.78
Pharmacist	39	15 (11.5)	24 (8.5)	
Nurse	129	44 (33.6)	85 (29.9)	
Medical assistant	54	17 (13.0)	37 (13.0)	
PPK	19	5 (3.8)	14 (4.9)	
Laboratory assistant	13	3 (2.3)	10 (3.5)	
**Work experience (year)**				
1–5	115	36 (27.5)	79 (27.8)	0.006
6–10	136	56 (42.7)	80 (28.2)	
11–15	102	26 (19.8)	76 (26.8)	
16–20	38	7 (5.3)	31 (10.9)	
≥21	24	6 (4.6)	18 (6.3)	
**Experience handling patients with COVID-19**				
Yes	331	99 (75.6)	232 (81.7)	0.45
No	84	32 (24.4)	52 (18.3)	
**Team building**				
Yes	135	44 (33.6)	91 (32.0)	0.003
No	280	87 (66.4)	198 (68.0)	
**Comorbidity**				
Present	81	23 (28.4)	58 (71.6)	0.56
Absent	334	108 (32.3)	226 (67.7)	
**PHQ-9 result**				
Depression	199	59 (45.0)	140 (49.3)	<0.001
No depression	216	72 (55.0)	144 (50.7)	
**GAD-7 result**				
Anxiety	54	32 (24.4)	22 (7.7)	0.026
No anxiety	361	99 (75.6)	262 (92.3)	

PHQ-9 - Patient Health Questionnaire-9

GAD-7 - Generalized Anxiety Disorder-7

Table 2 shows the results of the simple and multiple logistic regression analyses of the factors associated with low resilience among the participants. The simple logistic regression analysis revealed that female sex, work experience of ≤10 years, depression, anxiety and lack of leader appreciation and leader support were significantly associated with low resilience (P<0.05). The multiple logistic regression analysis included the variables with P-values of <0.25 in the simple logistic regression analysis: number of children and previous experience handling COVID-19 cases.

**Table 2:** Logistic regression analyses of the factors associated with low resilience among the participants (N=415).

Variable	Simple logistic regression			Multiple logistic regression		
	Crude OR	95% CI	P-value	Adjusted OR	95% CI	P-value
**Sex**						
Male	REF					
Female	1.69	1.00-2.84	0.05	1.87	1.05-3.32	0.033
**Work experience (year)**						
≤10	1.86	1.19-2.89	0.006	1.94	1.20-3.16	0.007
>10	REF					
**Depression**						
None	REF					
Depression	3.06	1.96-4.78	<0.001	2.53	1.46-4.41	<0.01
**Anxiety**						
None	REF					
Anxiety	3.63	1.16-11.32	0.026	1.47	0.42-5.13	0.540
**Leader support**						
Supportive	REF					
Not supportive	1.93	1.24-2.99	0.003	1.94	0.94-4.02	0.073

The final multiple logistic regression model revealed that three factors were significantly associated with low resilience: female sex, work experience of ≤10 years and depression. The female participants were nearly twice as likely to exhibit low resilience compared with their male counterparts (OR=1.87; 95% CI=1.05–3.32; P=0.033). Furthermore, the participants with ≤10 years of work experience had a two-fold increased likelihood of having low resilience compared with those with more experience (OR=1.94; 95% CI=1.2–3.16; P=0.007). Additionally, the participants with depression were twice more likely to have low resilience than those without depression (OR=2.53; 95% CI=1.46–4.41; P<0.001).

## Discussion

This study revealed that the prevalence of low resilience among primary HCWs in Kuala Lumpur was 31.6%. This figure is slightly higher than the prevalence reported in a metaanalysis that included healthcare professionals from 41 studies across 16 countries, which was 26%.^1^ Another meta-analysis demonstrated that the global prevalence of low resilience among the general population and health professionals during the COVID-19 pandemic was 27.0%.^3^ Notably, this study is the first of its kind to assess the prevalence of low resilience among primary HCWs in Kuala Lumpur. The results underscore that low resilience is common among primary HCWs in this region. This research was conducted during the post-COVID-19 period, and the findings highlight the dynamic nature of resilience, with certain populations experiencing more pronounced resilience at specific times and locations. Consequently, it is crucial to conduct longitudinal studies to examine the trajectory of resilience across the lifespan.

In this study, the primary factors contributing to low resilience among the primary HCWs were being a woman, having <10 years of work experience and experiencing depression. These findings align with those of other studies.^4,6^ Our study revealed that the female HCWs were twice more likely to exhibit low resilience than their male counterparts. This is consistent with a study conducted in Italy on the psychosocial impact of resilience among HCWs during the COVID-19 pandemic. The findings clearly demonstrate a significant relationship between low resilience and female sex.^4^ The study also revealed a significant connection between female sex and an increased risk of anxiety. Individuals with negative emotional states often exhibit lower psychological resilience. Notably, a systematic review conducted in Iran highlighted that female healthcare providers with anxiety disorders during the COVID-19 pandemic were especially vulnerable to experiencing stress symptoms. This underscores the need for targeted support for female healthcare professionals, who face unique stressors related to their sex and the challenges of balancing caregiving responsibilities with their professional obligations, making them more susceptible to anxiety than their male counterparts.^8^

Female HCWs have been shown to draw on social support as a key factor in managing stress, particularly when compared with their male counterparts. This reliance may be influenced by societal norms and cultural expectations. Social support is essential for reducing psychological distress and fostering positive emotions among HCWs.^4^ Addressing these challenges will necessitate systemic changes within healthcare organisations and broader societal shifts towards gender equality. Providing support, resources and opportunities for women to flourish can enhance their resilience and overall well-being.

A study conducted in Turkiye examined the impact of the COVID-19 outbreak on physicians’ psychological resilience. It revealed that physicians with 15 years of experience demonstrated higher psychological resilience. This aligns with our finding that the HCWs with over 10 years of experience had greater resilience than those with less experience. Although the studies did not explore the reasons behind this trend, it is plausible that more experienced HCWs have developed effective coping strategies to navigate the challenges of their profession.^6^

A recent global survey found that older adults showed higher resilience during the COVID-19 pandemic. This could be attributed to their enhanced ability to savour life experiences. Understanding this link can help to develop better coping strategies for all age groups.^9^ Experienced older adult HCWs have a deep understanding of their limitations, boundaries and self-care needs. Therefore, interventions and support systems must be customised for HCWs at different career stages. For instance, early-career professionals can benefit from mentorship programmes and skill development, while experienced professionals may find value in leadership training and peer support groups.^10,11^

In our study, the HCWs with depression had significantly lower levels of resilience. Another study that assessed anxiety, depression and psychological resilience among physicians working during the COVID-19 outbreak reported that the levels of depression and anxiety were significantly lower in physicians with greater psychological resilience.^6^ An Australian study conducted in 2020 during the COVID-19 pandemic explored changes in resilience among adults and their relationships with physical activity, depression, anxiety and stress. The findings revealed significant associations between resilience and psychological distress, highlighting the importance of understanding and promoting resilience during challenging times.^12^ Healthcare organisations should acknowledge the powerful influence of mental health issues, such as depression, on workforce resilience and prioritise it to offer vital support and resources.

### Limitations

Our study has certain limitations. The study participants were recruited from only eight selected public primary care clinics in four health administrative zones in Kuala Lumpur, out of over 1000 clinics throughout the country. A larger sample size would provide more representative data and enhance the study’s statistical power. The study utilised a cross-sectional design, offering an overview of resilience levels at a single period. Additionally, this study focused on HCWs in W.P. Kuala Lumpur, who have better facilities than those in suburban settings. Consequently, the factors identified in this study may not be relevant to and may reflect different populations. To address these limitations, future research can help to enhance the validity, reliability and generalisability of findings on resilience among public primary HCWs in other states or the entire country.

Our research underscores the critical need to regularly assess the resilience and depression levels of primary HCWs. A 2025 scoping review found that HCWs were at a significantly higher risk of developing mental illnesses such as depression and anxiety than the general population. The review emphasised that routine mental health assessments, including psychoeducation, psychotherapy and resilience-building interventions, are effective in improving well-being and reducing psychological distress.^13^ This is essential for developing stronger resilience to prevent burnout and reduce its detrimental impact on patient safety and care quality. Examining resilience among HCWs holds vast potential to improve individual well-being, enhance organisational effectiveness and ultimately elevate the standard of patient care in healthcare settings. By investing in resilience-building initiatives, healthcare organisations can foster a more robust workforce capable of meeting the demands of modern healthcare delivery. Examples of resilience-building interventions are structured training programmes (e.g. mindfulness-based stress reduction), teambuilding exercises and institutional support systems. Another comprehensive review identified various strategies that can effectively foster psychological resilience, such as cognitive-behavioural therapy, mindfulness and peer support networks. It also stressed the need for organisational support and leadership engagement to sustain these interventions.^14^

## Conclusion

The high prevalence of low resilience among primary HCWs necessitates urgent action. Factors such as being a woman, having ≤10 years of work experience and experiencing depression are linked to low resilience. A targeted resilience-building programme is crucial to enhance the wellbeing, job satisfaction and overall performance of primary HCWs. By recognising and addressing low resilience, healthcare organisations can substantially improve primary HCWs’ resilience, job satisfaction and quality of life, ultimately enhancing healthcare delivery and patient outcomes.

## Data sharing statement

The original contributions presented in the study are included in the article, further Inquiries can be directed to the corresponding author on reasonable request

**How does this paper make a difference in general practice?**

- Low resilience affects 31.6% of primary healthcare workers, highlighting a significant workforce issue.
- Identifies high-risk groups (female, <10 years’ experience, depression) for targeted interventions.
- It supports routine mental health screening and early identification in primary care settings.
- Informs the need for development of resilience-building programmes (e.g., mentorship, team support).
- Enhancing resilience may improve care quality, patient safety, and workforce sustainability
